# Variability in morphology and immunohistochemistry of Crohn’s disease-associated small bowel neoplasms: implications of Claudin 18 and Cadherin 17 expression for tumor-targeted immunotherapies

**DOI:** 10.1007/s00428-024-03896-4

**Published:** 2024-08-21

**Authors:** Mai Iwaya, Makoto Kodama, Keiko Abe, Kahoko Maeda, Tomoyuki Nakajima, Takeshi Uehara, Risa Nishio, Tetsuo Yamana, Robert Riddell, Hiroyoshi Ota

**Affiliations:** 1 Department of Laboratory Medicine, Shinshu University Hospital, 3-1-1 Asahi, Matsumoto, Nagano Japan; 2 Department of Pathology, Tokyo Yamate Medical Center, Tokyo, Japan; 3 Department of Laboratory Medicine, Shinshu University School of Medicine, Matsumoto, Japan; 4 Department of Coloproctology Center, Tokyo Yamate Medical Center, Tokyo, Japan; 5 Department of Pathology and Laboratory Medicine, Mount Sinai Hospital, Toronto, Canada; 6 Department of Laboratory Medicine and Pathobiology, University of Toronto, Toronto, Canada; 7 Department of Clinical Laboratory Sciences, School of Health Sciences, Shinshu University, Matsumoto, Japan

**Keywords:** Crohn’s disease, small bowel, Inflammatory bowel disease-associated carcinoma, dysplasia, Claudin 18 and Cadherin 17

## Abstract

*Aims:* Inflammatory bowel disease-associated colorectal carcinomas are known to have different morphology, immunoprofile, and genetic findings from sporadic colorectal carcinomas; however, little is known for Crohn’s disease-associated small bowel neoplasms (CD-SBNs). Cadherin 17 is a useful biomarker of adenocarcinomas with intestinal phenotype and recently reported as an ideal target for chimeric antigen receptor T-cells (CAR-T) therapy for gastrointestinal carcinoma. Claudin 18 is a cell adhesion protein, and Claudin18 isoform 2 (CLDN18.2) is frequently expressed at high levels in gastric-type adenocarcinoma. Zolbetuximab, a targeted monoclonal antibody, has been developed for CLDN18.2-positive gastroesophageal adenocarcinoma. We examined a series of CD-SBNs for both Cadherin 17 and Claudin 18, and also hypothesized that expression of Claudin 18 was associated with gastric phenotype.

*Methods and results:* We performed histological and immunohistochemical examinations on 25 CD-SBNs. Most of adenocarcinomas showed tubular morphology as seen in gastric carcinomas, whereas a subset of dysplasia was morphologically similar to that of the large bowel. Cadherin17 and Claudin 18 expression was identified in 93% and 57% CD-associated adenocarcinomas respectively. In Cadherin 17-positive CD-SBNs, frequent MUC5AC, MUC6, and Claudin18 expression was identified (61%, 57%, and 57%, respectively). Claudin 18-positive CD-SBNs showed significantly more MUC5AC and MUC6 expression than Claudin 18-negative CD-SBNs (*P* = 0.005, < 0.001 respectively).

*Conclusion:* In CD-associated small bowel adenocarcinomas, Cadherin 17 expression was frequently retained and Claudin 18 was frequently co-expressed. Claudin 18 had a positive correlation with the expression of gastric mucins. These results suggest that CD-associated small bowel adenocarcinomas may be candidates for Cadherin 17- and Claudin 18-targeted immunotherapies.

**Supplementary Information:**

The online version contains supplementary material available at 10.1007/s00428-024-03896-4.

## Introduction

Crohn’s disease (CD) and ulcerative colitis (UC) are the two most common forms of inflammatory bowel disease (IBD). Patients with IBD have an increased lifetime risk of developing colorectal adenocarcinoma (CRC). The molecular pathogenesis of colitis-associated carcinoma (CAC) is different than that of sporadic CRC, suggested that genomic changes linked to the effects of continuous inflammation and repeated mucosal injury in the setting of IBD [1, 2]. Although recent studies have shown that the risk of IBD patients developing CRC has decreased, probably as a result of better treatment and endoscopic surveillance [3–5], small bowel carcinomas in CD are still more likely to be found at an advanced stage since endoscopic surveillance is not standard for small bowel and it is often clinically difficult to distinguish strictures caused by inflammation from carcinoma. Two studies reported the histology of CD-associated small bowel adenocarcinomas [6, 7]; however, detailed histological analysis based on the current classification of IBD-associated dysplasia or adenocarcinoma have not been performed.

Cadherin-17 (aka liver-intestine cadherin) (CDH17) is a member of the cadherin superfamily and is a Ca2 + -dependent cell–cell adhesion molecule [8, 9] that is selectively expressed on enterocytes and goblet cells in the small and large bowel in human and mouse [10]. Several studies described CDH17 expression in adenocarcinoma of the digestive system [11, 12], and it is considered a useful biomarker of adenocarcinomas with intestinal phenotype [13, 14]. Recently, Feng et al. reported that CDH17 is an ideal target for chimeric antigen receptor T-cells (CAR-T) therapy for gastrointestinal carcinoma [15].

Claudins constitute a multigene transmembrane protein family of tight junctions that regulate paracellular transport and lateral diffusion of membrane lipids and proteins [16]. Claudin 18 (CLDN18) is a member of the CLDN family of cell surface proteins and CLDN18 isoform 2 (CLDN18.2) is normally expressed only in the stomach; Sahin et al. reported that CLDN18.2 is activated in a wide range of human malignancies, especially gastric, esophageal, and pancreatic adenocarcinoma [17]. Based on this, zolbetuximab, a targeted monoclonal antibody, was developed for patients with CLDN18.2-positive gastroesophageal adenocarcinoma [18–20] and CLDN18.2-specific CAR-T therapy has been recently developed [21, 22]. We have previously identified a higher rate of expression of CLDN18.2 in colitis-associated colorectal carcinomas with loss of intestinal markers such as SATB2, and the findings suggested colitis-associated colorectal carcinomas are promising candidates for CLDN18.2 targeted therapy [23].

In this study, we evaluate the histological characteristics of CD-associated small bowel dysplasia/adenocarcinoma and investigate the therapeutic potential of CDH17 and CLDN18 for tumor-targeted immunotherapies, and also whether expression of both CDH17 and CLDN18 are related to gastric differentiation using gastric MUC immunostains, MUC5AC, and MUC6.

## Methods

### Case selection

Study approval was obtained from the research ethics board at Shinshu University (5359, 22 November 2021) and Tokyo Yamate Medical Center (J-155, 7 September 2022).

Twenty-five consecutive lesions of surgically resected CD-SBN from 15 patients between 2012 and 2021 were retrieved from the surgical pathology archives at Tokyo Yamate Medical Center. Hematoxylin and Eosin (H&E) sections were reviewed by three gastrointestinal pathologists (MI, HO, and RR). Neoplastic lesions were classified into dysplasia and adenocarcinoma; one case showed that dysplastic glands invaded only into the muscularis mucosae; however, no obvious submucosal invasion was identified; this case was classified into intramucosal carcinoma (pTis). A total 14 adenocarcinomas and 11 dysplasias were evaluated in this study.

### Immunohistochemistry

At least one representative paraffin block of tumor was selected in each case for immunohistochemistry; if there was significant morphologic heterogeneity in a given case, multiple tumor blocks were selected as needed to adequately represent the entire tumor. Immunohistochemical staining was performed using commercially available antibodies with the immuno-enzyme polymer method (Histofine Simple Stain MAX PO Multi (Nichirei Biosciences, Tokyo, Japan) for MUC2, MUC5AC, MUC6, and SATB2, or Novolink Polymer Detection Systems (Leica, Wetzlar, Germany) for CDH17, CLDN18, and beta-catenin) with 3,3′-diaminobenzidine as the chromogen, or an automated slide preparation system (p53: VENTANA BenchMark ULTRA, Roche, Basel, Switzerland).

The following primary antibodies were used in accordance with the manufacturers’ instructions: CDH17 (clone: SP183; Cell Marque, Rocklin, CA, USA), CLDN18 (clone: EPR19203; Abcam, Cambridge, UK), MUC2 (clone CCP58, Agilent, Santa Clara, CA, USA), MUC5AC (clone: CLH2; Agilent), MUC6 (clone: CLH5; Novus Biologicals, Centennial, CO, USA), SATB2 (clone: EPNCIR130A; Abcam), beta-catenin (clone: EP35, Cell Marque) and p53 (clone: DO7, Agilent).Microsatellite-instability testing by immunohistochemistry for mismatch repair proteins (MMRs) (MLH1(clone: M1, Roche), MSH2 (clone: G219-1129, Roche), MSH6 (clone: SP93, Roche), and PMS2 (clone: A16-4, Roche) was conducted on an automated slide preparation system (VENTANA BenchMark ULTRA, Roche).

The extent of staining for CDH17, CLDN18, MUC2, MUC5AC, and MUC6 was scored semiquantitatively (no staining; < 10%; 10–25%; 26–50%; 51–75%; and 76–100%), and the maximum intensity was graded as negative, weak, moderate, or strong. For binary analyses, cases with 10% or more tumor cells showing moderate or strong intensity were considered positive (Supplemental Fig. 1). For CDH17 and CLDN18, cases with ≥ 40% [18] or 75% [19] tumor cells showing moderate or strong intensity were also noted which met the participation criteria of the CLDN18 clinical trials [18, 19]. To evaluate any a possible correlation with wnt pathway mutations with CDH17 or CLDN18 changes, beta-catenin staining was classified as membranous expression or nuclear expression. Expression of p53 was classified as wild type (variable weak to moderate staining) or mutant type (either diffuse strong staining or complete absence of staining). For any relationship with mismatch repair gene proteins MLH1, MSH2, MSH6, and PMS2, retained expression was defined as nuclear staining of any intensity within tumor cells, using infiltrating lymphocytes as a positive internal control. Deficient mismatch repair protein expression was defined as complete loss of expression of at least one of the 4 mismatch repair proteins. Two of the authors (MI and HO) reviewed the immunohistochemical stains and reached a consensus score.

**Fig. 1:**
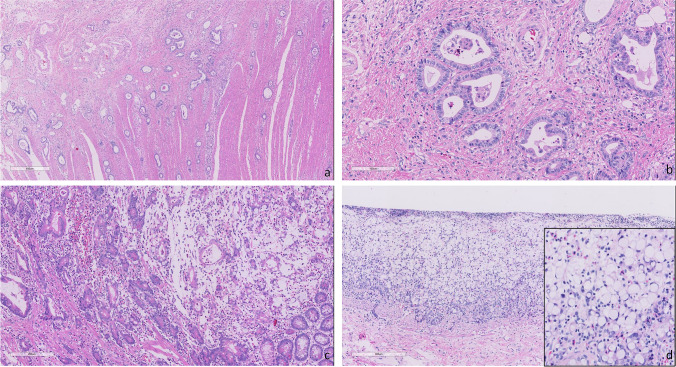
Histology of Crohn’s disease-associated small bowel adenocarcinomas. Low-power view of infiltrating pattern (**a**) and high-power view of tubular morphology (**b**). Tubular and mucinous morphology (**c**) with focal signet ring cell differentiation (**d**)

### Statistics

Chi-squared test or Fisher’s exact tests were used to characterize the relationship between categorical variables. Differences were considered significant at *P* < 0.05. All statistical analyses were performed with EZR (Saitama Medical Center, Jichi Medical University, Saitama, Japan), which is a graphical user interface for R (The R Foundation for Statistical Computing, Vienna, Austria).

## Results

### Study group characteristics

In fifteen patients, 12 (80%) patients had one carcinoma and 1 (7%) patient had 2 carcinomas for a total of 14 adenocarcinomas were identified. Eleven of 14 (79%) lesions were found in the ileum and 1 (7%) lesion was found in the jejunum, while in 2 (14%) lesions the precise location within the small bowel was not stated. Eleven of 14 (79%) lesions showed at least muscularis propria invasion and 5 (45%) of them were classified into pT4. Lymph node dissection was performed in 3 cases and no metastasis was identified.

Eight patients with adenocarcinoma had synchronous dysplasia. One patient had 2 foci of dysplasia and 7 patients had 1 focus; 5 foci were adjacent to adenocarcinoma. Two of 15 (13%) patients had 1 focus of dysplasia only and for a total of 11 dysplasias were identified. The median age of included patients was 50 years (range: 29–71 years); of the patients, 11 were male and 4 were female.

### Pathologic features and immunohistochemistry

#### Histology of adenocarcinoma

None of the cases showed conventional type colorectal carcinoma morphology which represents cribriform glands composed of epithelium with stratified long oval nuclei and occasional intraluminal necrosis. Thirteen of 14 (93%) cases showed similar morphology; invasive glands were composed of epithelium with cuboidal nuclei and abundant dense eosinophilic cytoplasm; nuclear stratification was not frequently seen. The features were similar to tubular adenocarcinoma of the stomach (Fig. 1); thus, we decided to subclassify tumor morphology by WHO classification of gastric cancer [24]. Two of them combined different morphology such as signet ring cell or poorly cohesive cellular histological components therefore subclassified into mixed adenocarcinoma (Fig. 1). The other 1 case had mucinous and poorly cohesive cellular histological components and subclassified into mixed adenocarcinoma.

#### Histology of dysplasia

**Fig. 2:**
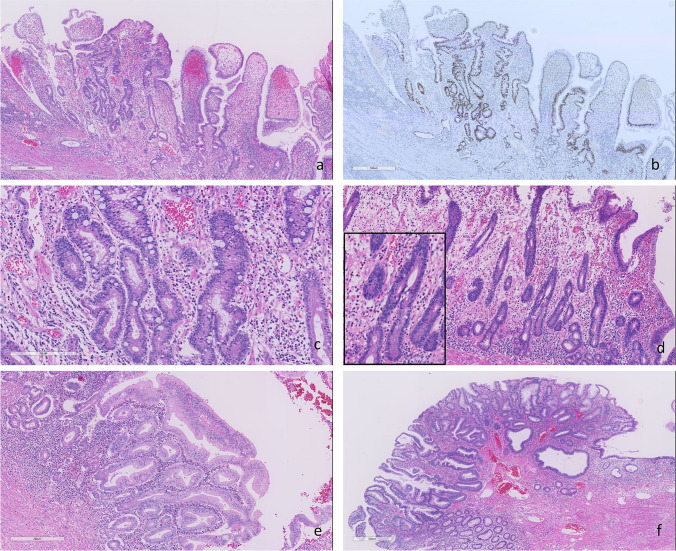
Histology of Crohn’s disease-associated small bowel dysplasias. Thickened villous appearance with irregular small glands (**a**), these glands are diffusely immunoreactive for p53 (mutant pattern) (**b**). Dystrophic goblet cells are seen in the small glands (high-power view of **a**) (**c**); (**a**–**c**; terminally differentiated (non-conventional type) dysplasia). Terminally differentiated dysplasia with mucin depletion (**d**). Serrated dysplasia (**e**). Adenomatous (conventional type) dysplasia (**f**)

Dysplasias showed similar morphology with IBD-associated dysplasia in large bowel and they were classifiable into conventional (adenomatous) and non-conventional morphology (24, 25). Four of 11 (36%) dysplasia showed conventional morphology, and three of them were adjacent carcinoma. Seven (64%)  lesions were subclassified into non-conventional dysplasia and one lesion showed serrated morphology, and the others showed terminally differentiated morphology and overlapping mucin depleted morphology was occasionally observed (Fig. 2).

A summary of the clinicopathological features of the cohort is shown in Table 1.

**Table 1:** Clinicopathological features of Crohn’s disease-associated small bowel neoplasms

Clinical and pathologic features	
No. of cases (no. of patients)	25 (15)
Age (median)	29–71(50)
Sex, male/female	11 / 4
Type of neoplasm (%)	
Adenocarcinoma	14 (56)
Dysplasia adjacent carcinoma	5 (20)
Dysplasia only	6 (24)
Adenocarcinomas	
Location (%)	
Jejunum	1 (7)
Ileum	11 (79)
Unknown	2 (14)
pT stage (%)	
is	1 (7)
1	2 (14)
2	3 (21)
3	3 (21)
4	5 (36)
pN stage (%)	
Negative	3 (21)
Positive	0 (0)
Unknown	11 (79)
Tumor subtypes (%)	
Tubular adenocarcinoma	11 (79)
Mixed adenocarcinoma	3 (21)
Dysplasia	
Subtypes (%)	
Conventional	4 (36)
Non-conventional	7 (64)
Low grade/high grade	11(100) / 0(0)

#### Immunohistochemistry

**Fig. 3:**
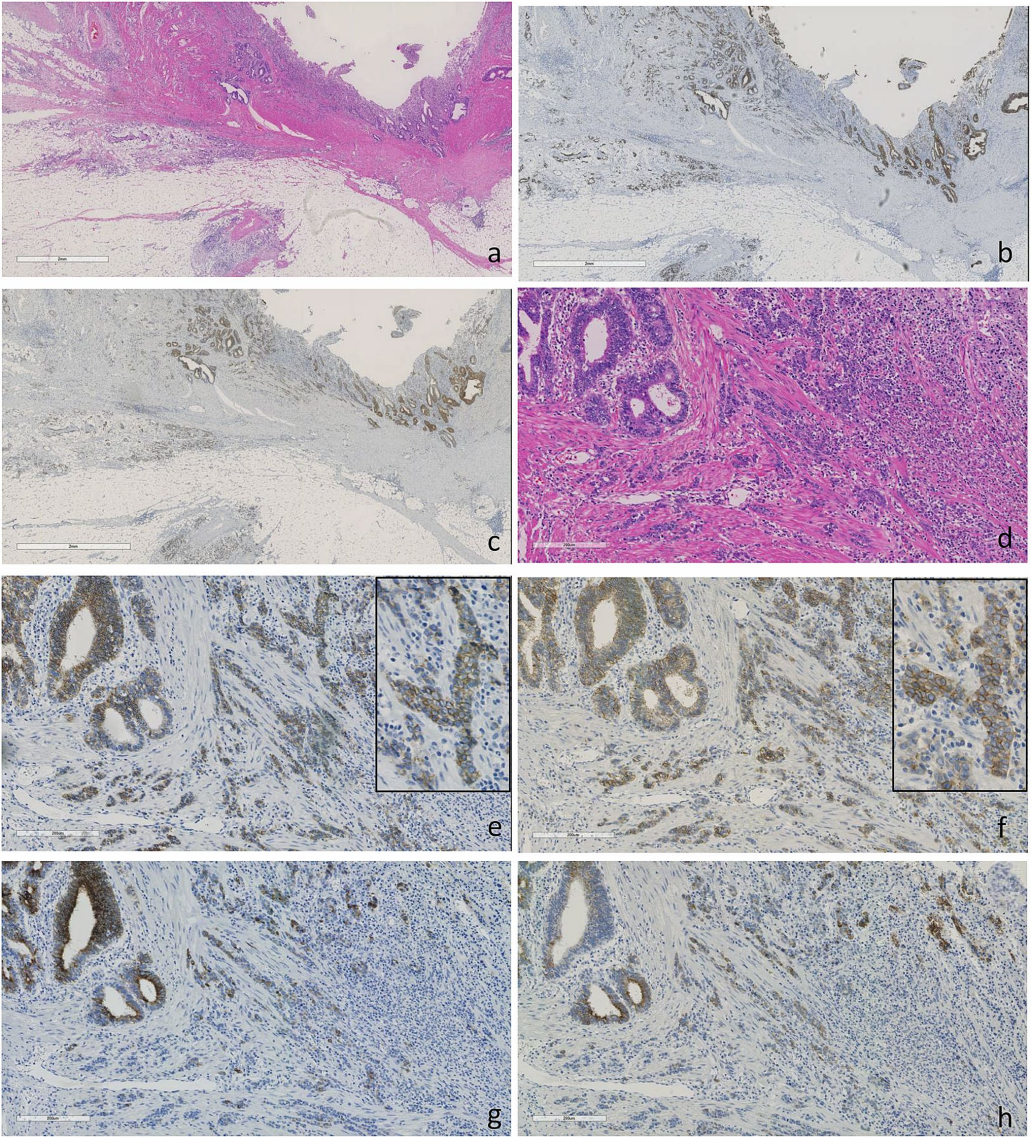
Low-power view of Crohn’s disease-associated small bowel adenocarcinoma (mixed (tubular and poorly cohesive) adenocarcinoma histology). **a** With diffuse cadherin 17 expression (**b**) and claudin 18 expression (**c**). High-power view H&E (**d**) with diffuse strong cadherin 17 expression (**e**), claudin 18 expression (**f**), MUC5AC expression (**g**), and MUC6 expression (**h**)

CDH17 expression was retained in thirteen of 14 (93%) CD-associated adenocarcinomas and ≥ 40% extent expression was seen in 12 cases (86%), 9 of them showed ≥ 75% extent expression. Eight of 14 (57%) CD-associated adenocarcinomas were positive for CLDN18, and 5 (36%) lesions showed ≥ 40% extent expression and 2 (14%) of them showed ≥ 75% extent expression. Seven of 13 (54%) CDH17-positive CD-associated adenocarcinomas were positive for CLDN18 (Fig. 3). In dysplasias, ten of 11 (91%) cases were positive for CDH17 and 6 of 11 (55%) were positive for CLDN18 (Table 2).

**Table 2:** Immunohistochemistry for Crohn’s disease-associated small bowel neoplasms

	Adenocarcinoma (%)	Dysplasia (%)
Number of cases	14	11
CDH17	13 (93)	10 (91)
CLDN18	8 (57)	6 (55)
MUC5AC	8 (57)	7 (64)
MUC6	8 (57)	6 (55)
MUC2	9 (64)	8 (73)
SATB2	1 (7)	2 (18)
P53 (mutant)	4 (29)	6 (55)
beta-catenin (nuclear expression)	1 (7)	2 (18)
MMR (deficient)	0 (0)	0 (0)

CDH17 was positive in 23 of 25 (93%) of CD-SBNs. In CDH17-positive CD-SBNs, 61% were positive for MUC5AC, 57% were positive for MUC6, and 57% were positive for CLDN18. Fourteen of 25 (56%) CD-SBNs were positive for CLDN 18 and between CLDN18-positive CD-SBNs and CLDN18-negative SBNs; CLDN18-positive CD-SBNS showed significantly more MUC5AC and MUC6 expression than CLDN18-negative CAC (*P* = 0.005, < 0.001 respectively). Two cases of superficially invasive carcinomas showed surface predominant MUC5AC expression and deep layer MUC6 expression, similar expression to that seen in normal gastric mucosa (Supplemental Fig. 2). There was no significant difference in the immunoprofile of CDH17, MUC2, beta-catenin, and p53 mutant ratio between CLDN18-positive CD-SBNs and CLDN18-negative SBNs. Three of 25 (12%) CD-SBNs (1 conventional type invasive carcinoma and 2 conventional type dysplasias; 1 dysplasia was adjacent to SATB2 positive invasive carcinoma) were SATB2 positive and all SATB2-positive CD-SBNs showed CDH17 expression, whereas no CLDN18 expression was identified. All lesions were MMR proficient (Table 3).

**Table 3:** Immunoprofile of Crohn’s disease-associated neoplasms with and without CLDN18 expression

Pathological features	CLDN18 positive (%)	CLDN18 negative (%)	*p*
Tumor subtypes			
Adenocarcinoma	8	6	
Dysplasia	6	5	
Immunohistochemistry (%)			
CDH17	13 (93)	10 (91)	1
MUC5AC	12 (86)	3 (27)	0.005
MUC6	13 (93)	1 (9)	< 0.001
MUC2	9 (64)	8 (73)	1
P53 (mutant)	4 (29)	6 (55)	0.241
beta-catenin (nuclear expression)	1 (7)	2 (18)	0.565
SATB2	0 (0)	3 (27)	0.072
MMR (deficient)	0 (0)	0 (0)	1

## Discussion

Here, we evaluated the tumor morphology and immunohistochemical expression of CDH17 and CLDN18 in 25 CD-SBNs and potential relationships with gastric differentiation as indicated by MUC5AC and MUC6 immunohistochemistry, wnt pathway mutations, and mismatch repair gene protein immunohistochemistry. Our results showed that regardless of adenocarcinoma or dysplasia, CDH17 expression was retained in most CD-SBNs and CLDN18 expression was seen in 56% of CD-SBNs with a strong association with the expression of gastric mucins. No association was identified on p53, beta-catenin, or MMR status with CDH17 and CLDN18 expression. CD-associated small bowel adenocarcinomas were morphologically more similar to gastric carcinomas than colorectal carcinomas; however, dysplasia morphology was similar to that seen in the colitic mucosa. We also found some CD-SBNs showed SATB2 expression that is normally expressed in the large intestinal epithelium and considered a relatively specific marker for colorectal adenocarcinomas [25].

CDH17 is a member of the cadherin superfamily which is selectively expressed in the epithelial cells of small and large bowel and considered a useful biomarker of adenocarcinomas with intestinal phenotype [12–14]. Su et al. reported that diffuse CDH17 expression was seen in 96% of colorectal carcinomas and 56% of gastric adenocarcinomas; however, they noted most gastric cases showed focal or scattered staining patterns [12]. Interestingly, we found 92% of CD-SBNs were CDH17 positive and 91% of them showed ≥ 40% extent with moderate or strong membranous expression, and approximately half of them co-expressed gastric-type mucins, such as MUC5AC and MUC6 with frequently CLDN18. The findings suggest that CD-SBNs retained intestinal phenotype, that gastric differentiation can co-exist. We also found that 57% of CD-associated adenocarcinoma showed CLDN18 expression and the ratio was higher than that of colitis-associated colorectal carcinomas in our previous study [23]. In the study, we used the same immunohistochemical antibody and cutoff criteria and demonstrated that CLDN18 expression was seen in 27% of colitis-associated colorectal carcinomas with an association of MUC5AC expression, while without significant association with MUC6 status [23]. The positive ratio of CLDN18 was also higher than that in the study on small bowel adenocarcinomas recently reported by Arpa et al. They noted that 28% of small bowel adenocarcinomas were immunoreactive for CLDN18 with a positive correlation of MUC5AC expression, using cutoff values of ≥ 1% at any intensity[26]. The findings might be affected by background disease since they used not only CD-associated adenocarcinomas, with including sporadic and celiac disease cases. In our study, 3 CD-SBNs (12%) showed SATB2 expression. Similar less frequent expression of SATB2 in CD-associated small bowel adenocarcinomas was reported by Neri et al. [27]. They found 20% of small bowel adenocarcinomas showed SATB2 expression; however, the positive ratio was lower in CD-associated adenocarcinomas (12%) than in sporadic or celiac disease-associated adenocarcinomas, suggesting CD-associated small bowel adenocarcinomas are less likely to have large bowel differentiation. Whitcomb et al. reported that CD-associated small bowel adenocarcinomas were more likely to show MUC5AC and MUC6 expression than sporadic small bowel adenocarcinomas [7]. In clinical practice, pyloric gland metaplasia and gastric foveolar metaplasia are frequently seen in small bowel in CD patients_._ Previous studies showed the frequent MUC5AC and CK7 expression in small bowel mucosa in CD patients [28] and the frequent MUC5AC expression in colonic mucosa in IBD patients [29]. Although the precise molecular mechanism remains undefined, aberrant expression of CLDN18 and gastric-type mucins in the setting of CD might be linked to the effects of ongoing inflammation and repeated mucosal injury.

Initially, we attempted to classify adenocarcinomas into one of five morphological subtypes (conventional, mucinous, serrated, low-grade tubuloglandular (LGTG), and others) which we previously used in IBD-associated colorectal carcinomas [23, 30, 31]; however, small bowel CD-associated adenocarcinomas were morphologically different from IBD-associated colorectal carcinomas and none of our cases showed conventional colorectal morphology. Most of our cases showed morphology similar to tubular adenocarcinoma of the stomach, as in the previous study [7]. Considering the gastric-type immunoprofile and morphology, adenocarcinoma of the small bowel is more likely to show pronounced gastric differentiation than adenocarcinoma of the large bowel in IBD.

We also evaluated the immunophenotype and morphology of CD-associated small bowel dysplasias and found that CD-associated small bowel dysplasia showed frequent CLDN18 and gastric-type mucins expression, with rarely SATB2 expression, with both conventional (adenomatous) and non-conventional morphology which has been described in IBD-associated dysplasia in the large bowel [32, 33]. In this cohort, 64% of cases were subclassified into non-conventional dysplasia and we found serrated, terminally differentiated and mucin depleted morphology. This finding is in accordance with the previous study by Simpson et al. who reported dysplasias in CD-associated small bowel and described histology as follows; “adenomatous,” “saw-tooth or serrated pattern,” and “dysplastic Paneth’s cells and basal cell change,” and noted that the features were similar with dysplasias seen in UC patients [34]. These findings indicated that CD-associated small bowel dysplasia show similar immunoprophile with adenocarcinomas; frequent CLDN18 and gastric type mucins expression, with rarely SATB2 expression. However, dysplasias shared a similar histology with colitic dysplasia, whereas adenocarcinomas were histologically similar with gastric adenocarcinomas. Due to the small number of cases examined in our study, further studies in a larger cohort are needed for detailed histological evaluation in CD-associated small bowel dysplasias.

By finding retained CDH17 expression and frequent CLDN18 expression, our study indicates the possibility of widened treatment options in CD-associated small bowel carcinoma patients. Feng et al. recently reported that they developed a llama-derived nanobody, VHH1-driven CAR-Ts targeting CDH17 and demonstrated that the VHH1-CAR-T cells (CDH17CAR-Ts) eradicated CDH17 expressing neuroendocrine tumor and gastrointestinal cancers such as gastric, pancreatic, and colorectal cancers in tumor xenograft or autochthonous mouse models. They noted CDH17CAR-T did not cause histological damage in normal intestinal cells [15]. Although further investigation is warranted for clinical implementation, most CD-associated small bowel adenocarcinomas may be a candidate for CDH17 CAR-T therapy. Furthermore, we previously reported that frequent immunohistochemical CLDN18 (clone: EPR19203) expression in colitis-associated colorectal carcinomas and confirmed CLDN18-positive colorectal carcinomas only expressed *CLDN 18.2* by RT-PCR [23]. The finding of frequent expression of CLDN18 in CD-associated small bowel carcinomas has implications for a targeted anti-claudin 18.2 antibody such as zolbetuximab therapy. Recently, phase 3 zolbetuximab trial in patients with CLDN18.2 positive (defined as ≥ 75% of tumor cells showing moderate or strong membranous CLDN18 staining), HER2 negative, locally advanced unresectable or metastatic gastric or gastroesophageal junction (ClinicalTrials.gov Identifier: SPOTLIGHT; NCT03504397) resulted in significantly prolonged progression-free survival and overall survival [19]. We found 14% of CD-associated small bowel adenocarcinomas match the participation criteria of the trial and the findings indicate that zolbetuximab therapy might be effective for a subset of CD-associated small bowel carcinoma which are still more likely to be found at the advanced stage.

In conclusion, we demonstrated that CDH17 was frequently retained even approximately half of CDH17-positive CD-SBNs showed gastric mucin expression and CLDN18 expression was frequently co-expressed. CLDN18 expression had a positive correlation with the expression of gastric mucins. CD-associated small bowel adenocarcinoma histology was different from colitis-associated colorectal carcinomas; however, a subset of small bowel dysplasia was morphologically similar to that of the large bowel. These results suggest that CD-associated small bowel adenocarcinomas may be candidates for CDH17- and CLDN18-targeted immunotherapies.

## Supplementary Information

Below is the link to the electronic supplementary material.

Supplementary file1 Figure 1. Immunohistochemical scoring of Cadherin 17 and Claudin 18 expression. Examples of tumors scored as having absent (a), weak (b), moderate (c), or strong (d) membranous expression are shown (Claudin18 immunostain) Nb (TIF 15298 KB)

Supplementary file2 Figure 2. Histology of superficially invasive adenocarcinoma (a), high power view (b). MUC5AC expression is seen mainly in the superficial to middle layers of the lamina propria (c), and MUC6 expression is seen mainly in the middle to deep layers of the lamina propria (c). Cadherin 17 expression pattern is similar with MUC5AC (e), and Claudin 18 expression is similar with MUC6 (f) (TIF 21874 KB)

## Data Availability

The datasets generated during and/or analyzed during the current study is available from the corresponding author on reasonable request.
