# Supplementary figures and images for: Variability in morphology and immunohistochemistry of Crohn’s disease-associated small bowel neoplasms: implications of Claudin 18 and Cadherin 17 expression for tumor-targeted immunotherapies
Source: Virchows Arch. 2024 Aug 21;486(3):595–603. doi: 10.1007/s00428-024-03896-4 (PMC11950054; doi:10.1007/s00428-024-03896-4)

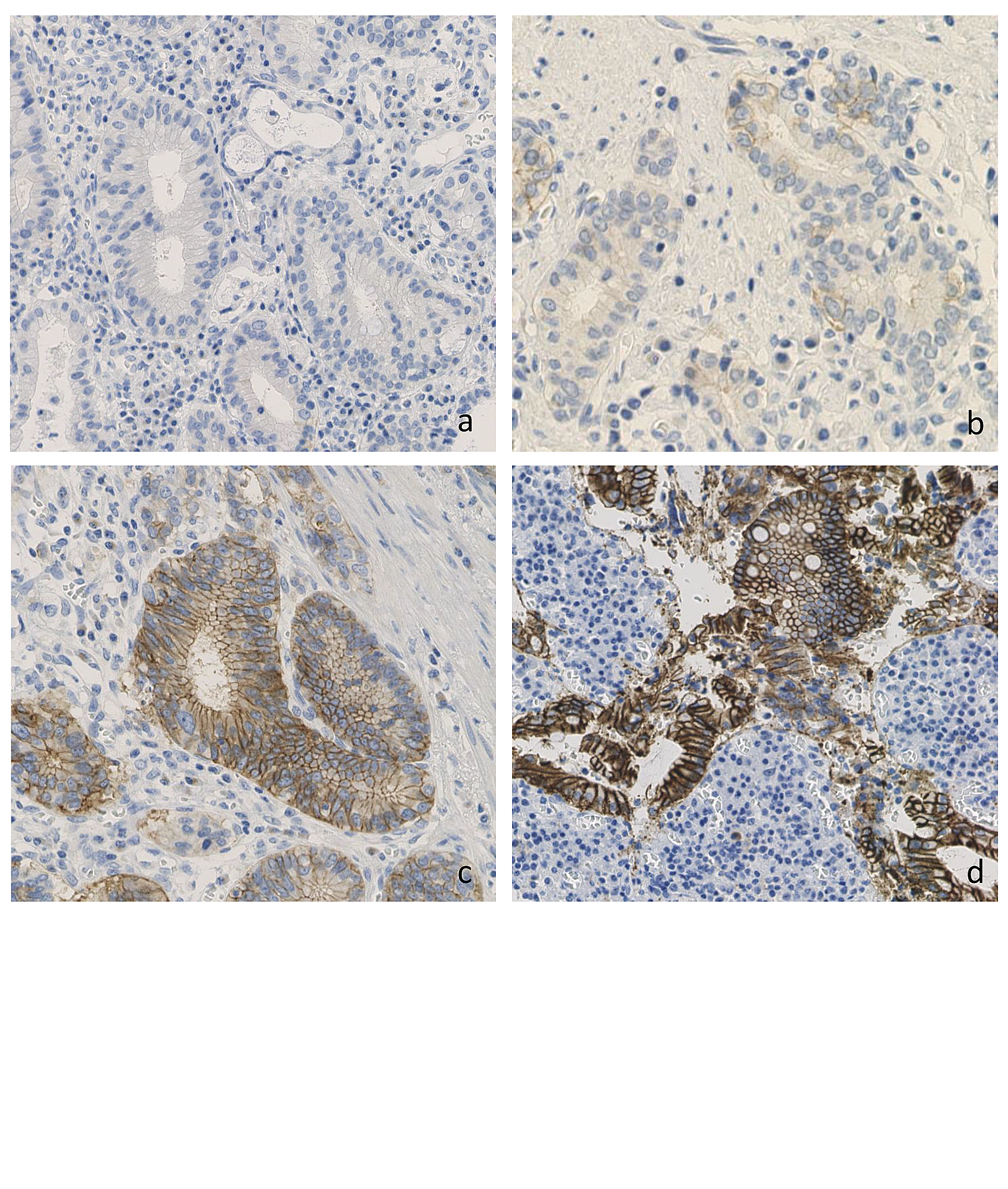

Supplement: Supplementary file 1 — Supplementary file1 Figure 1. Immunohistochemical scoring of Cadherin 17 and Claudin 18 expression. Examples of tumors scored as having absent (a), weak (b), moderate (c), or strong (d) membranous expression are shown (Claudin18 immunostain) Nb (TIF 15298 KB) [file 428_2024_3896_MOESM1_ESM.tif]

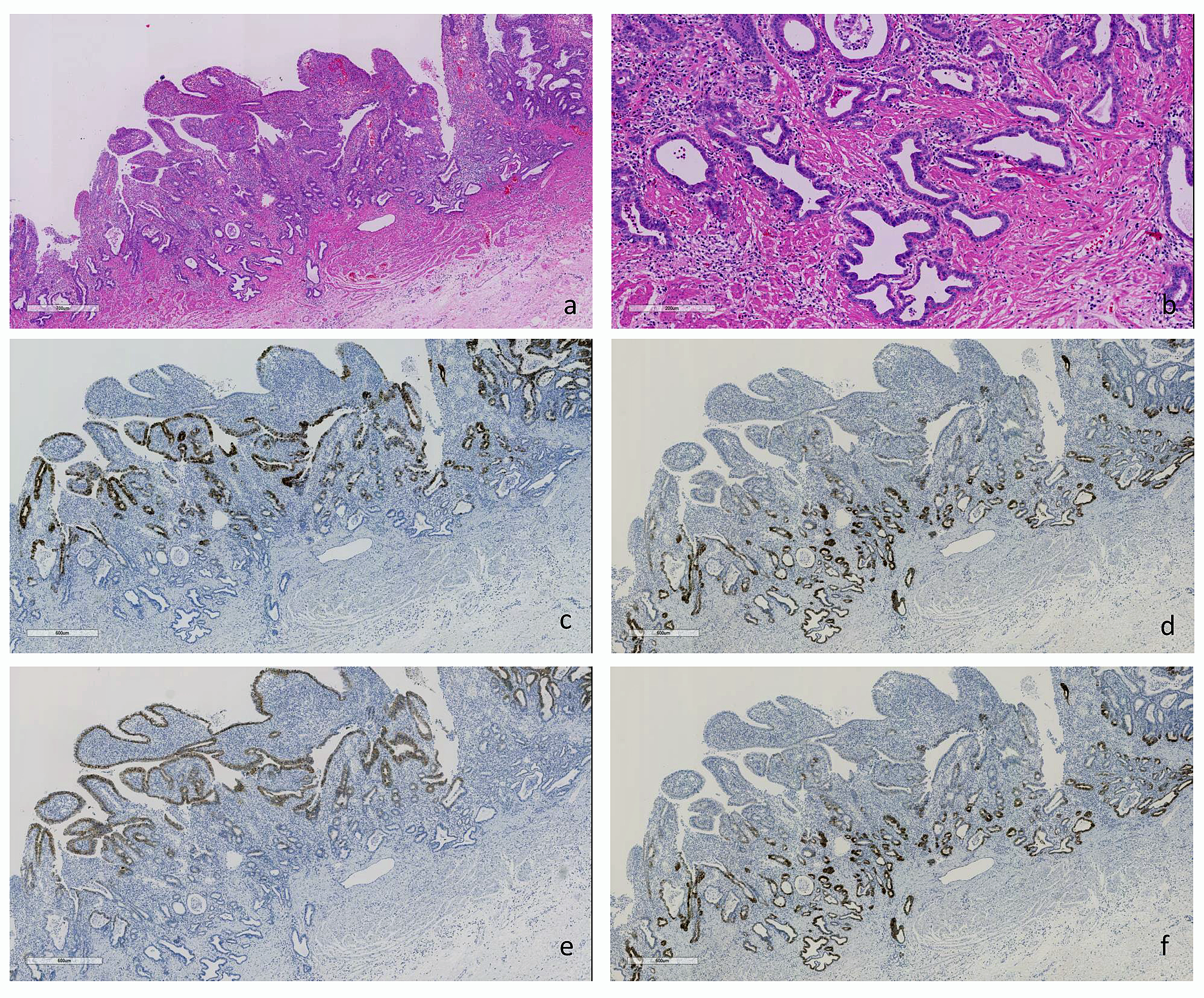

Supplement: Supplementary file 2 — Supplementary file2 Figure 2. Histology of superficially invasive adenocarcinoma (a), high power view (b). MUC5AC expression is seen mainly in the superficial to middle layers of the lamina propria (c), and MUC6 expression is seen mainly in the middle to deep layers of the lamina propria (c). Cadherin 17 expression pattern is similar with MUC5AC (e), and Claudin 18 expression is similar with MUC6 (f) (TIF 21874 KB) [file 428_2024_3896_MOESM2_ESM.tif]
